# Morpholinoethoxy-Substituted Cationic Metal-Free and Metallo Phthalocyanines: In Vitro Photodynamic Therapy Activities, PDT-Induced ROS Level Measurements, and Cellular Death Mechanism

**DOI:** 10.1021/acsbiomedchemau.5c00137

**Published:** 2025-06-26

**Authors:** Muge Serhatli, Seyma Isik, Ayfer Kalkan, Mukaddes Özçeşmeci, Esin Hamuryudan, Özge Can

**Affiliations:** † Climate Change and Life Sciences, Biotechnology Research Group, TUBITAK Marmara Research Center, Kocaeli 41470, Turkey; ‡ Department of Medical Biotechnology, Graduate School of Health Sciences, Acibadem Mehmet Ali Aydinlar University, Istanbul 34752, Turkey; § Faculty of Science and Letters, Department of Chemistry, 52971 Istanbul Technical University, 34469 Istanbul, Turkey; ∥ Department of Biomedical Engineering, Faculty of Engineering and Natural Sciences, Acibadem Mehmet Ali Aydinlar University, Istanbul 34752, Turkey

**Keywords:** cancer, cationic, photodynamic therapy, phthalocyanine, reactive oxygen species

## Abstract

In this study, morpholinoethoxy-attached cationic tetra-substituted phthalocyanines (Pcs), including metal-free (**HQH**_**2**_ **Pc**), zinc (**HQZnPc**), and indium (**HQInPc**) derivatives, were synthesized following established protocols. Their structures were confirmed by using standard spectroscopic techniques. The photodynamic therapy (PDT) efficacy of these compounds was evaluated against head, neck, and colon cancer cell lines. Reactive oxygen species (ROS) levels induced by PDT with cationic Pcs were quantified by using dichlorodihydrofluorescein diacetate. To elucidate the mechanisms of action, ROS generation was assessed at two distinct time points: 30 min (immediate response) and 24 h (delayed response) post-PDT. The cellular death mechanisms induced by Pc-mediated PDT in cancer cell lines were investigated using fluorescence staining with Apopxin Green, CytoCalcein Violet 450, and 7-AAD to differentiate apoptotic and necrotic pathways and provide insights into the mode of cell death. The results indicated that the Pcs exhibited minimal cytotoxicity in the absence of light, confirming their safety as photosensitizers. Cationic Pcs, particularly **HQZnPc**, showed high PDT-induced cytotoxicity and ROS production, primarily inducing apoptosis in cancer cell lines, with FaDu cells exhibiting the highest sensitivity. These results highlight **HQZnPc**’s strong potential for cancer therapy and underscore the need for further research into its delivery and mechanisms in complex tumor models.

## Graphical abstract

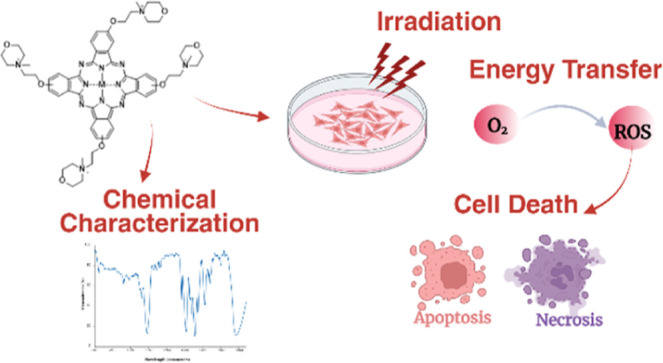

## 1 Introduction

Cancer is a complex and diverse group of diseases characterized by the uncontrolled growth of abnormal cells, which can invade nearby tissues and spread to other parts of the body through the blood and lymphatic systems.^1^ The complexity of cancer has driven the development of various FDA-approved treatment modalities, including chemotherapy, radiotherapy, and immunotherapy. Among these, photodynamic therapy (PDT) has emerged as a highly promising option due to its precision in targeting malignant cells while minimizing harm to surrounding healthy tissues. PDT is particularly valuable for certain cancer types, especially those resistant to standard treatments, and remains a focus of extensive research aimed at optimizing its application and efficacy.^2,3^

PDT relies on three essential components: a light source, a photosensitizing agent, and molecular oxygen, which interact to produce cytotoxic reactive oxygen species (ROS) and singlet oxygen, leading to the targeted destruction of tumor cells.^4–6^ The first step of PDT involves the administration of a photosensitizer, which preferentially accumulates in the tumor tissues. Upon exposure to light at a specific wavelength, the photosensitizer is activated and transitions to an excited triplet state, triggering photochemical reactions primarily with molecular oxygen.^7^ Photophysical activation of ground-state oxygen in PDT occurs via two distinct pathways. The type I pathway involves the transfer of electrons or protons from the excited photosensitizer to molecular oxygen, producing ROS that causes localized cellular damage. Alternatively, the type II pathway involves direct energy transfer to oxygen, generating singlet oxygen.^8^ The short lifespan (10–350 ns) and limited diffusion range (∼10–55 nm) of singlet oxygen ensure that its cytotoxic effects are highly localized, minimizing harm to nearby healthy tissues.^9,10^ PDT induces cell death through various mechanisms, including apoptosis, necrosis, or a combination of both, depending on factors such as light dosage, subcellular localization of the photosensitizer, and the type of cancer cells.^11^ Lower light doses in PDT typically trigger apoptosis, a controlled and programmed form of cell death, whereas higher doses tend to induce necrosis, likely due to the suppression of caspase enzymes and other proteins involved in the apoptotic pathway.^12,13^ The precision and versatility of PDT highlights its potential as a minimally invasive and highly targeted cancer treatment.

Phthalocyanines (Pcs), synthetic macrocyclic compounds, have garnered significant attention as photosensitizers for PDT due to their advantageous photophysical properties, particularly their high molar extinction coefficient in the red Q-band region.^14,15^ In their excited triplet state, Pcs generate 26–30 kcal/mol of energy, which is sufficient for efficient singlet oxygen production, requiring only 23 kcal/mol. Pcs offer several key advantages, including selective accumulation in tumors, low toxicity in the absence of light, efficient singlet oxygen generation, fluorescence for imaging, prolonged triplet-state lifetimes, and excellent photostability. Their prominent absorption peak in the red-light region (∼680 nm) is particularly beneficial for targeting tumors in deeper tissues, addressing a critical challenge in treating cancers in hard-to-reach areas.^14,16^ These properties make Pcs highly effective for both clinical and research applications in PDT.^17^ The chemical flexibility of Pcs enables functional group modifications to improve water solubility and enhance interactions with target cells, thereby optimizing their effectiveness in PDT.^18,19^ Additionally, the incorporation of various metal ions into the central cavity of Pcs significantly influences their photophysical properties. Metallo-Pcs exhibit varying photoreduction efficiencies, with zinc-containing Pcs demonstrating the highest efficiency, followed by those with aluminum, magnesium, and iron cores.^20,21^

Determining the biological properties of Pcs is very important for their medical applications. For this purpose, interactions of Pcs with biological materials have been the subject of various studies, and quite successful results have been obtained from studies involving Pcs substituted with groups that interact easily with biological molecules such as DNA/BSA. Morpholine, which is a cyclic structure containing nitrogen and oxygen, can be given as an example of these substituted groups. Morpholine and its derivatives, whose activities have been approved by many pharmaceutical applications, have been identified as analgesics and anesthetics.^22–24^ They are also of interest due to their anti-inflammatory, antidepressant, and antitumor activities.^25,26^ Studies have shown that the binding of morpholine to the Pc structure modifies the physicochemical properties and amphiphilic nature of Pcs, thus facilitating their potential applications in biology and medicine.^27–30^

According to the current state of the art, the complexation of Pcs with different ions can affect the PDT activity. Even open-shell paramagnetic ions can reduce the activity.^31^ Therefore, the photodynamic activities of Pcs with different central metal ions need to be further investigated. In addition to the metal ion located in the Pc center, substituents are also important in PDT activity. Some substituents, especially amino groups, have been reported to affect the fluorescence and ROS production. Moreover, quaternary ammonium groups further enhance the interaction of drugs via electrostatic interaction, which is beneficial for cellular uptake.^32^ Inspired by both the investigation of the effect of different metal ions and the findings obtained from the studies on morpholine groups in the literature, this paper focuses on morpholinoethoxy group-substituted cationic Pcs having different central metal ions. In our previous study, we tested the *in vitro* phototoxicity and cytotoxicity behaviors of quaternized Pcs against the cervical cancer cell line called HeLa in order to evaluate their suitability for cancer treatment by the PDT method.^33–35^ The binding properties of cationic Pcs to CT-DNA were also investigated by fluorescence and UV–vis spectrophotometric methods. Here, in this study, *in vitro* PDT activities of tetra-substituted cationic metal-free (**HQH**_**2**_ **Pc**), zinc (**HQZnPc**), and indium Pcs (**HQInP**c) carrying morpholinoethoxy groups were evaluated against different cancer cell lines: submaxillary salivary gland epidermoid carcinoma (A253), colorectal adenocarcinoma (HT29), and pharyngeal squamous carcinoma (FaDu). Additionally, PDT-induced ROS generation and cell death mechanisms activated in response to Pc-mediated PDT were evaluated.

## 2 Results and Discussion

### 2.1 Characterization of Cationic Phthalocyanines

Tetra-substituted cationic Pcs (**HQH**_**2**_ **Pc**, **HQZnPc**, and **HQInPc**) were synthesized in parallel with the previous study.^35^ First, neutral Pcs were obtained by cyclotetramerization of phthalonitrile compound in the presence of metal salt and 8-diazabicyclo[5.4.0]­undec-7-ene (DBU) base (no metal salt was used in the synthesis of metal-free Pcs). Then, by treating the obtained neutral Pcs with excess methyl iodide, cationic derivatives (**HQH**_**2**_ **Pc**, **HQZnPc**, and **HQInPc**) were prepared with 60–75% yields (Figure 1). While neutral Pcs were soluble in many organic solvents, their cationic derivatives were only soluble in DMF, DMSO, and water, as expected.

**Figure 1:**
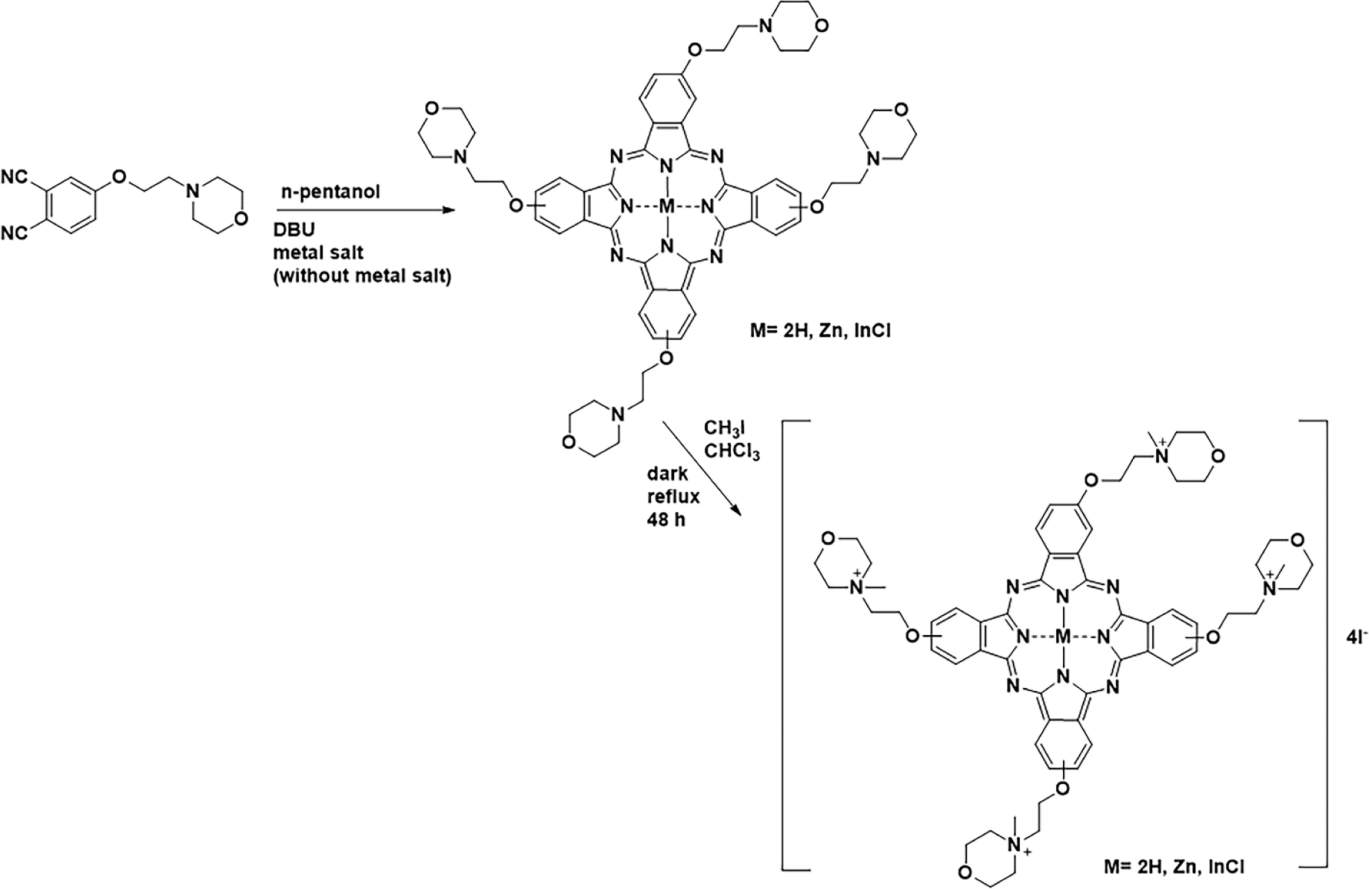
Preparation of tetra-substituted cationic phthalocyanines.

The structures of the obtained cationic Pcs were confirmed by spectroscopic methods. In the FT-IR spectra of cationic Pcs (**HQH**_**2**_ **Pc**, **HQZnPc**, and **HQInPc**), aromatic stretching vibrations were observed around 3000 cm^–1^, while aliphatic stretching vibrations were detected in the range of 2870–2950 cm^–1^. In the IR spectrum of metal-free Pcs (**HQH**_**2**_ **Pc**), vibrations belonging to the inner-NH groups were additionally detected at 3289 cm^–1^.

In the ^1^H NMR spectra of cationic Pcs taken in DMSO-*d*_6_, aromatic protons were observed in the range 9.50–7.80 ppm, and aliphatic protons were in the range of 5.10–3.30 ppm. In addition, protons belonging to methyl groups were detected around 3.50 ppm. Pcs were characterized by characteristic B and Q bands in the UV–vis spectrum. In the spectra of cationic Pcs (**HQH**_**2**_ **Pc**, **HQZnPc**, and **HQInPc**) taken in DMF, Q bands were recorded in the range of 670–700 nm, and B bands were recorded between 340 and 360 nm (Figure 2).

**Figure 2:**
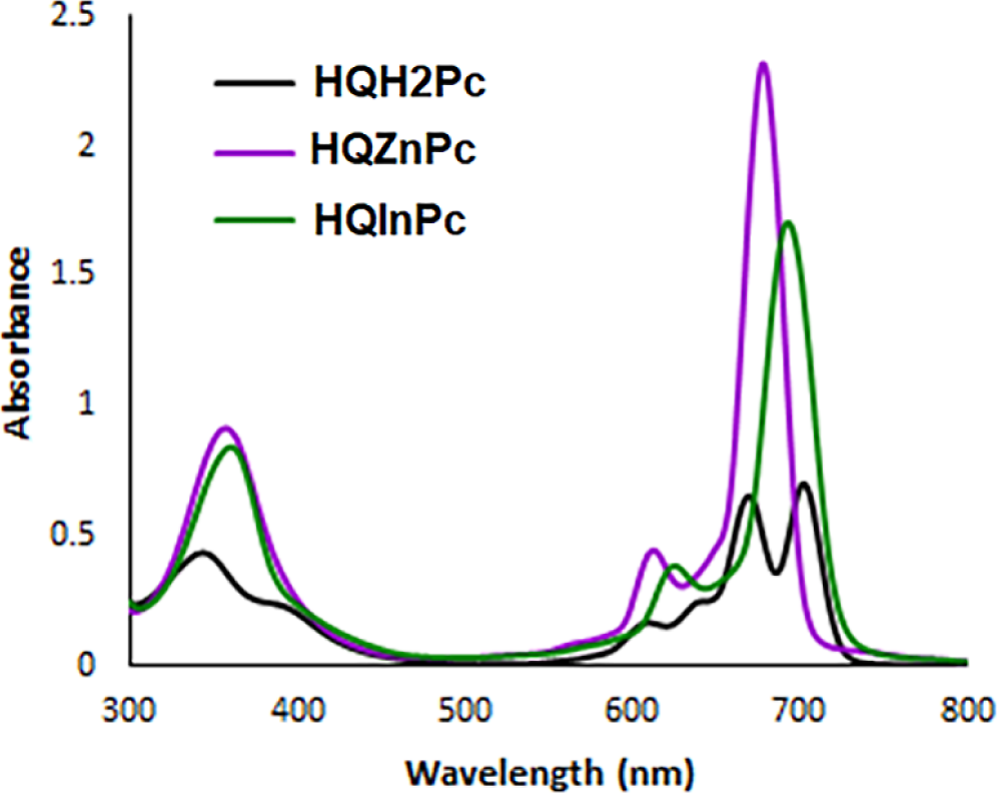
UV–vis absorption spectra of **HQH**_**2**_ **Pc**, **HQZnPc**, **and HQInPc** in DMF (1 × 10^–5^ M).

### 2.2 In Vitro Studies

#### 2.2.1 PDT Activities against Head, Neck, and Colon Cancer Cell Lines

Low cytotoxicity in the absence of light is an essential characteristic of an effective photosensitizer in PDT applications. The colorimetric WST-1 assay was utilized to evaluate the dark- and PDT-induced cytotoxicity of the morpholinoethoxy-containing metallo- and (**HQInPc** and **HQZnPc**) metal-free (**HQH**_**2**_ **Pc**) Pcs on the A253, FaDu, and HT29 cells (Figure 3). The results indicated that, at concentrations up to 10 μM, the Pc molecules exhibited no significant cytotoxicity in the absence of light across all three cell lines, confirming that these molecules are nontoxic in dark conditions, a fundamental requirement for effective photosensitizers. PDT using the metallo Pc molecules (**HQInPc** and **HQZnPc**) resulted in substantial cell death across all cell lines, with cell viability decreased to below 50%, whereas metal-free Pc (**HQH**_**2**_ **Pc**) did not induce a comparable reduction across all tested cell lines and concentrations (Figure 3A,D,G), indicating limited photodynamic efficacy. In A253 cells, treatment with **HQInPc** at 10 μM reduced the viability to 44% (Figure 3B), while **HQZnPc** at 1 μM achieved a reduction to 49% (Figure 3C). In HT29 cells, viability decreased to 36% and 45% following treatment with **HQInPc** (10 μM) and **HQZnPc** (1 μM), respectively (Figure 3H,I). The most pronounced phototoxic effect was observed in FaDu cells, with **HQInPc** (10 μM) and **HQZnPc** (0.1 μM) reducing viability to 20% and 46%, respectively (Figure 3E,F). Notably, HQZnPc demonstrated a greater cytotoxicity than **HQInPc** at lower concentrations, suggesting superior photodynamic efficiency. These results demonstrate the promise of **HQZnPc** as a more effective photosensitizer, particularly in PDT-responsive cell lines, such as FaDu.

**Figure 3:**
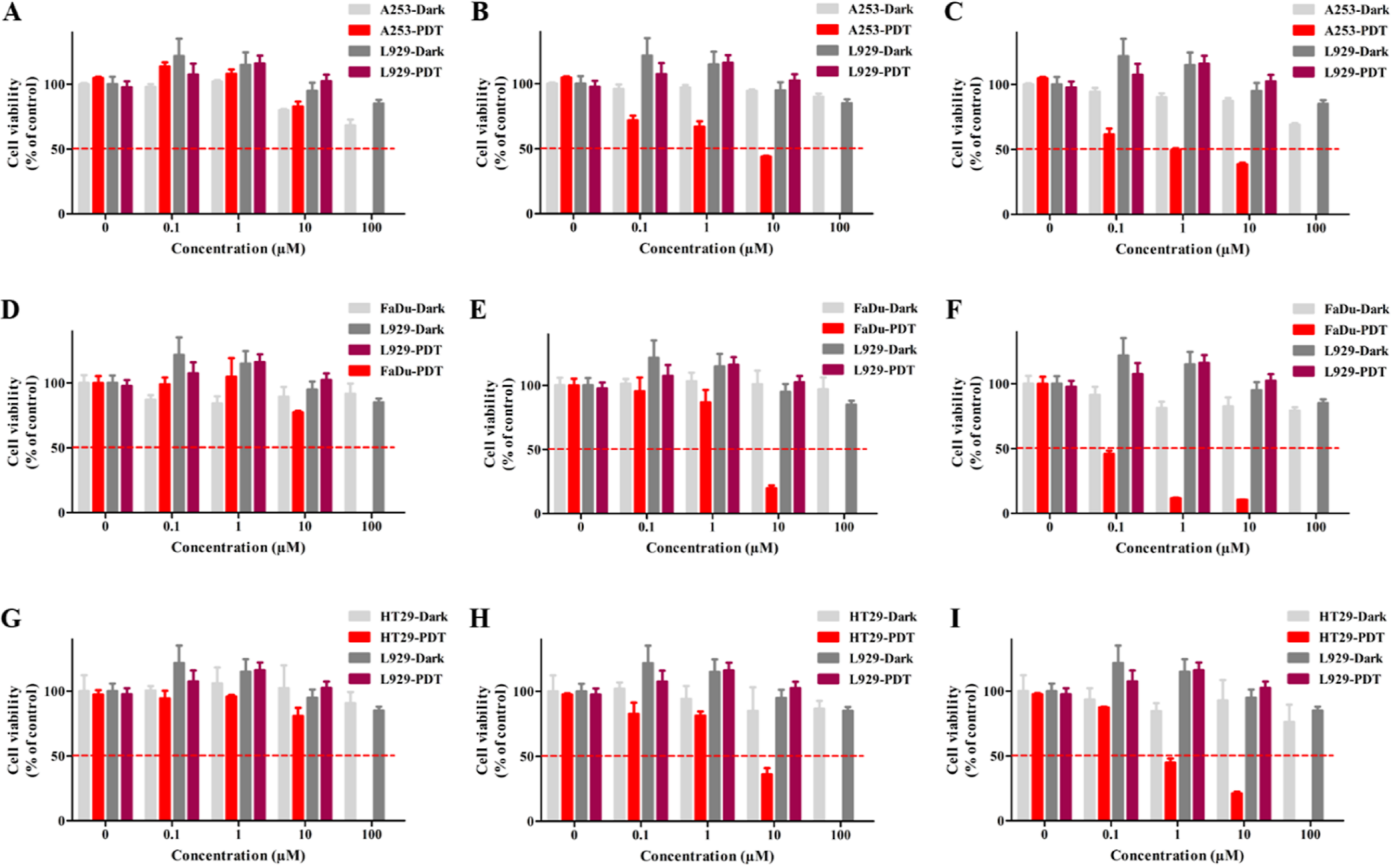
Dark- and PDT-induced cytotoxicity of Pcs. PDT with the Pcs molecules, (A,D,G) **HQH**_**2**_ **Pc**, (B,E,H) **HQInPc**, and (C,F,I) **HQZnPc**, induced a dose-dependent cytotoxicity with varying degrees, without affecting cell viability in the absence of light in (A–C) A253, (D–F) FaDu, and (G–I) HT29 cells. **HQH2Pc-PDT led** to moderate cytotoxicity only at high concentrations; in contrast, **HQZnPc** caused significant phototoxicity even at lower concentration, in all the studied cell lines. Results are shown as means ± SD of three independent experiments. *p < 0.05, **p < 0.01, and ***p < 0.001.

In order to assess the specificity and safety profile of the photosensitizers, the noncancerous fibroblast cell line L929 was utilized as a negative control. Importantly, L929 cells exhibited no detectable cytotoxicity under either dark conditions or following photodynamic treatment with any of the Pc derivatives across all tested concentrations. The absence of phototoxic effects in the noncancerous L929 cell line reinforces the selective nature of the photosensitizing compounds toward cancerous cells, underscoring their potential for targeted PDT with minimal off-target effects.

Axially or core-substituted closed-shell Pcs, especially AlPc, SiPc, and ZnPc derivatives, are known to be suitable for applications in cancer PDT.^36^ The main advantages of closed-shell transition metal Pcs for PDT applications lie in their easily tunable optical properties and triplet state formation. These are responsible for the effective singlet oxygen production required for the photodynamic effect. Especially recently, interesting studies have been conducted on the biological and anticancer applications of polycationic zinc Pcs (ZnPcs).^37^ This is because cationic groups provide electrostatic interactions with the negatively charged cell membrane and enhance phototoxicity through better interaction with cellular components. In our study, consistent with the literature, **HQZnPc** displayed better cell death than **HQH**_**2**_ **Pc** and **HQInPc** in all cells studied at both low and high doses due to the closed-shell effect.

### 2.3 PDT-Induced ROS Production

The generation of ROS upon activation of the photosensitizer by light triggers irreversible oxidation of critical cellular components, leading to tissue damage and constituting the primary mechanism of PDT. Molecular oxygen is essential in this process as ROS-mediated cellular damage primarily arises from energy transfer between excited Pc molecules and nearby oxygen molecules.

PDT-induced ROS levels were measured using dichlorodihydrofluorescein diacetate (DCFDA) at two distinct time points: 30 min (immediate) and 24 h (delayed) post-PDT. **HQH**_**2**_ **Pc**-mediated PDT produced minimal ROS in FaDu (Figure 4D) and HT29 (Figure 4G) cells, with no significant changes in A253 cells (Figure 4A) compared to the untreated control. In contrast, the metallo-Pcs **HQInPc** (Figure 4B,E,H) and **HQZnPc** (Figure 4C,F,I) generated significantly higher ROS levels across all cell lines, with HQZnPc inducing the most substantial ROS production, particularly in FaDu cells (Figure 4F). Additionally, ROS production exhibited a clear dose- and time-dependent increase, peaking at 24 h post-PDT. Zn-centered Pc consistently generated the highest ROS levels across all cell lines, highlighting the enhanced efficacy of **HQZnPc**. The strong correlation between ROS levels and cytotoxicity confirms that PDT-induced cell damage is primarily driven by ROS generation. The higher delayed ROS levels compared with immediate levels indicate prolonged ROS activity, leading to sustained oxidative stress within the cells. These findings underscore the significant effect of the central metal ion in Pc molecules on ROS formation and its critical contribution to the effectiveness of PDT. In the case of metallo-Pcs, it is clear that **HQZnP**c has the highest level due to the closed-shell effect. When the studies conducted—in the literature, especially on metal-free and ZnPcs—were examined, it was reported that ZnPcs gave more positive results, as expected.^38,39^ The results obtained from this study are consistent with those of studies in the literature.

**Figure 4:**
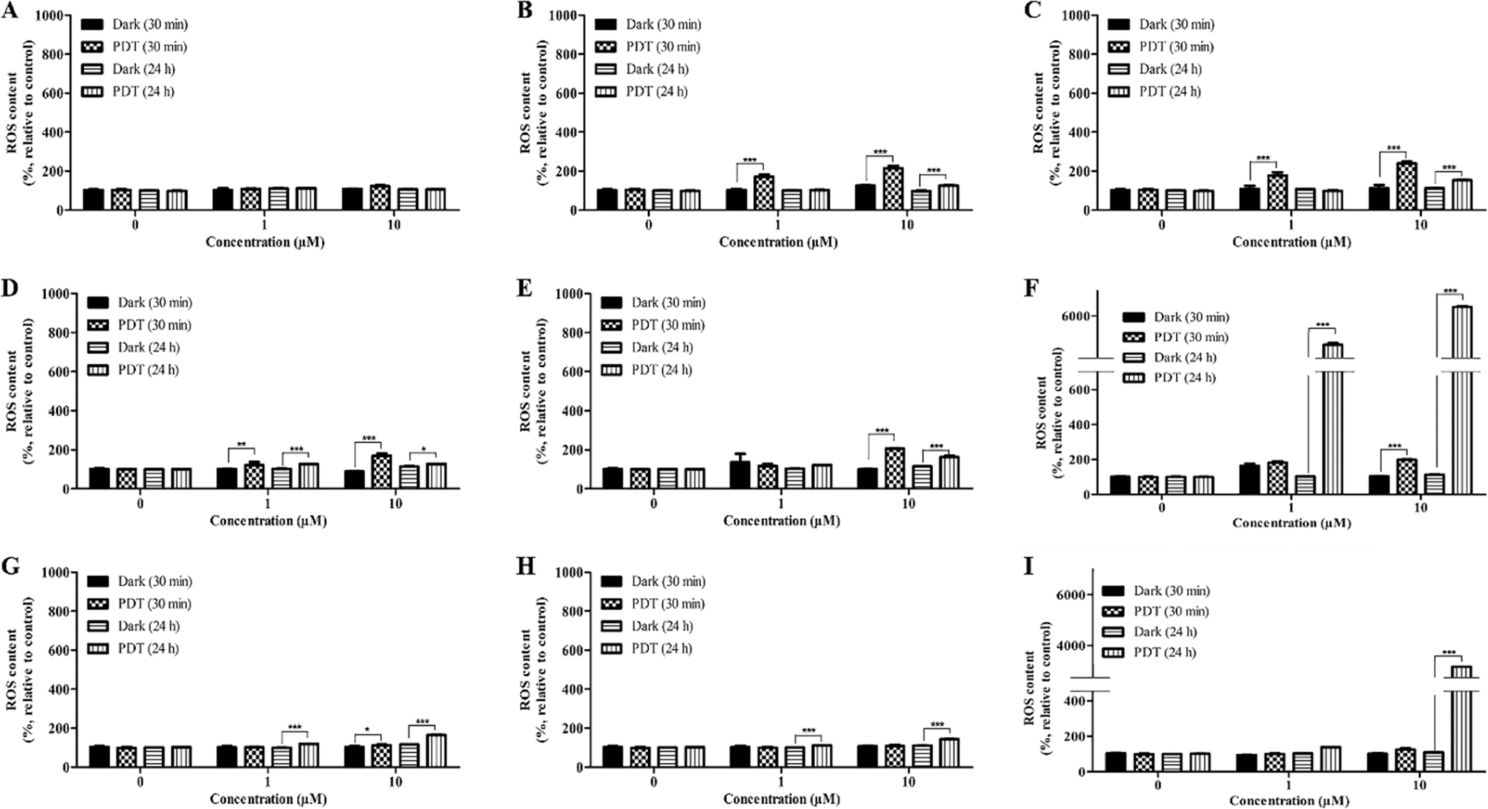
PDT-induced ROS production mediated by the Pcs molecules, (A,D,G) **HQH**_**2**_ **Pc**, (B,E,H) **HQInPc**, and (C,F,I) **HQZnPc**, in (A–C) A253, (D–F) FaDu, and (G–I) HT29 cells. Results are means ± SD of three independent experiments. *p < 0.05, **p < 0.01, and ***p < 0.001. PDT light dose: 20 J cm^–2^.

### 2.4 Cellular Death Mechanism (Apoptosis/Necrosis)

The cellular death mechanisms induced by Pc-mediated PDT in cancer cell lines were examined by using fluorescence staining with Apopxin Green, CytoCalcein Violet 450, and 7-AAD. PDT-induced ROS production drives cellular damage, resulting in apoptosis, necrosis, or both. A hallmark of early apoptosis is the externalization of phosphatidylserine (PS) from the inner to outer plasma membrane leaflet. Apopxin Green binds to these exposed negatively charged PS residues, identifying apoptotic cells (green), while CytoCalcein Violet 450 marks viable cells (blue), and 7-AAD stains necrotic cells (red). The results indicated that PDT with the metal-free Pc (**HQH**_**2**_ **Pc**) exhibited minimal cytotoxicity, inducing only a small fraction of apoptotic cells, consistent with its limited therapeutic potential (Figure 3). In contrast, PDT with metallo-Pcs (**HQInPc** and **HQZnPc**) induced significant cell death across all tested cancer cell lines, predominantly through apoptotic pathways. In A253 (Figure 5) and HT29 cells (Figure 7), apoptosis was the primary mode of cell death, with necrosis occurring at lower but notable levels. Similarly, the majority of FaDu cells (Figure 6) exhibited apoptotic cell death, with necrosis observed in a negligible fraction.

**Figure 5:**
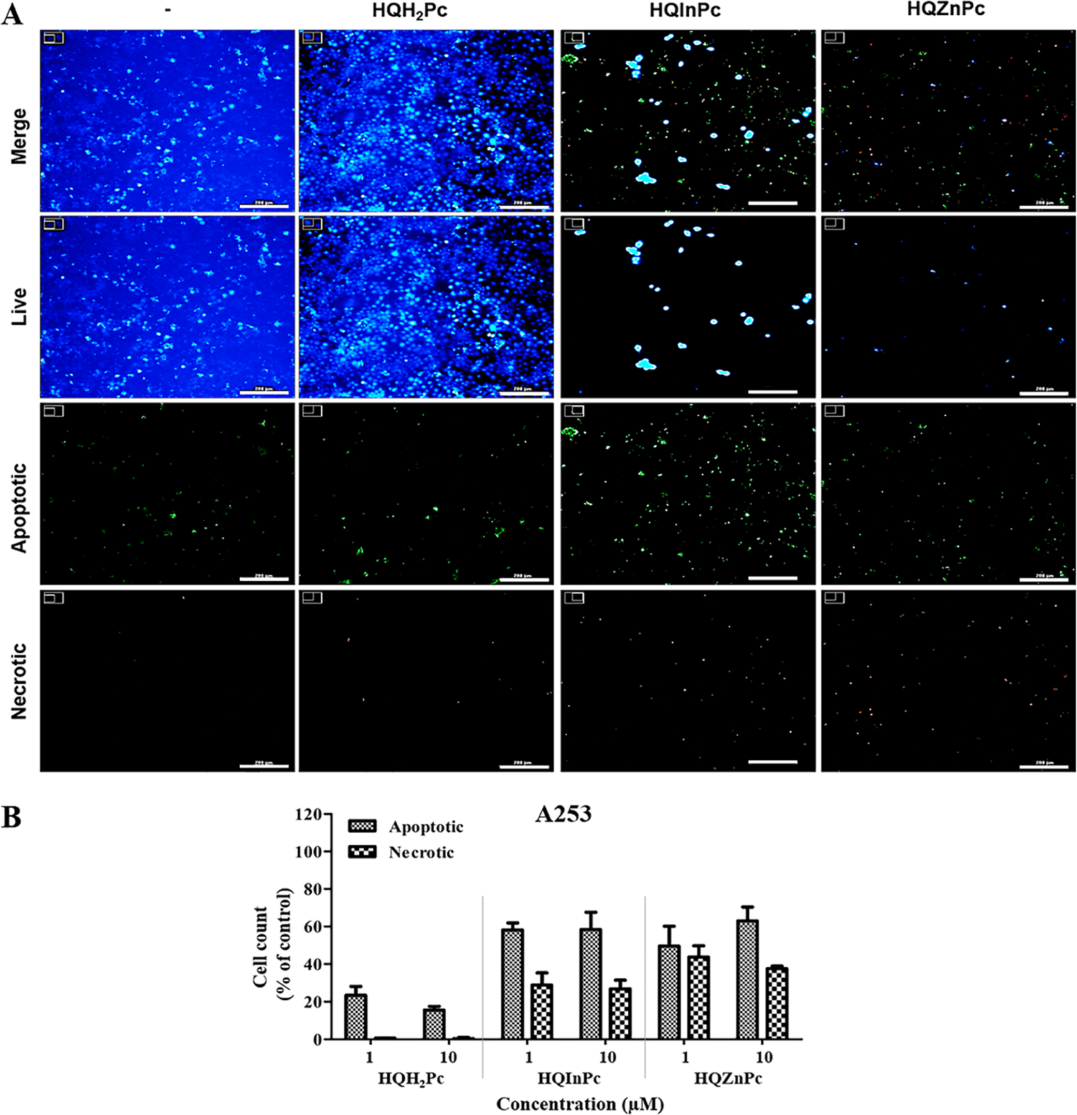
PDT-induced cellular death mechanism on A253 cells mediated by **HQH**_**2**_ **Pc**, **HQInPc**, and **HQZnPc** molecules. (A) Fluorescence microscopy images of A253 cells stained to identify live, apoptotic, and necrotic populations 24 h after PDT. Cells were treated with 10 μM HQH_2_Pc, HQInPc, or HQZnPc and irradiated with a light dose of 20 J/cm^2^. Live cells were stained with CytoCalcein Violet 450 (blue), apoptotic cells with Apoxin Green (green), and necrotic cells with 7-AAD (red). Scale bar: 200 μm. (B) Quantification of apoptotic and necrotic cell populations following treatment, presented as percentages relative to the total cell population.

**Figure 6:**
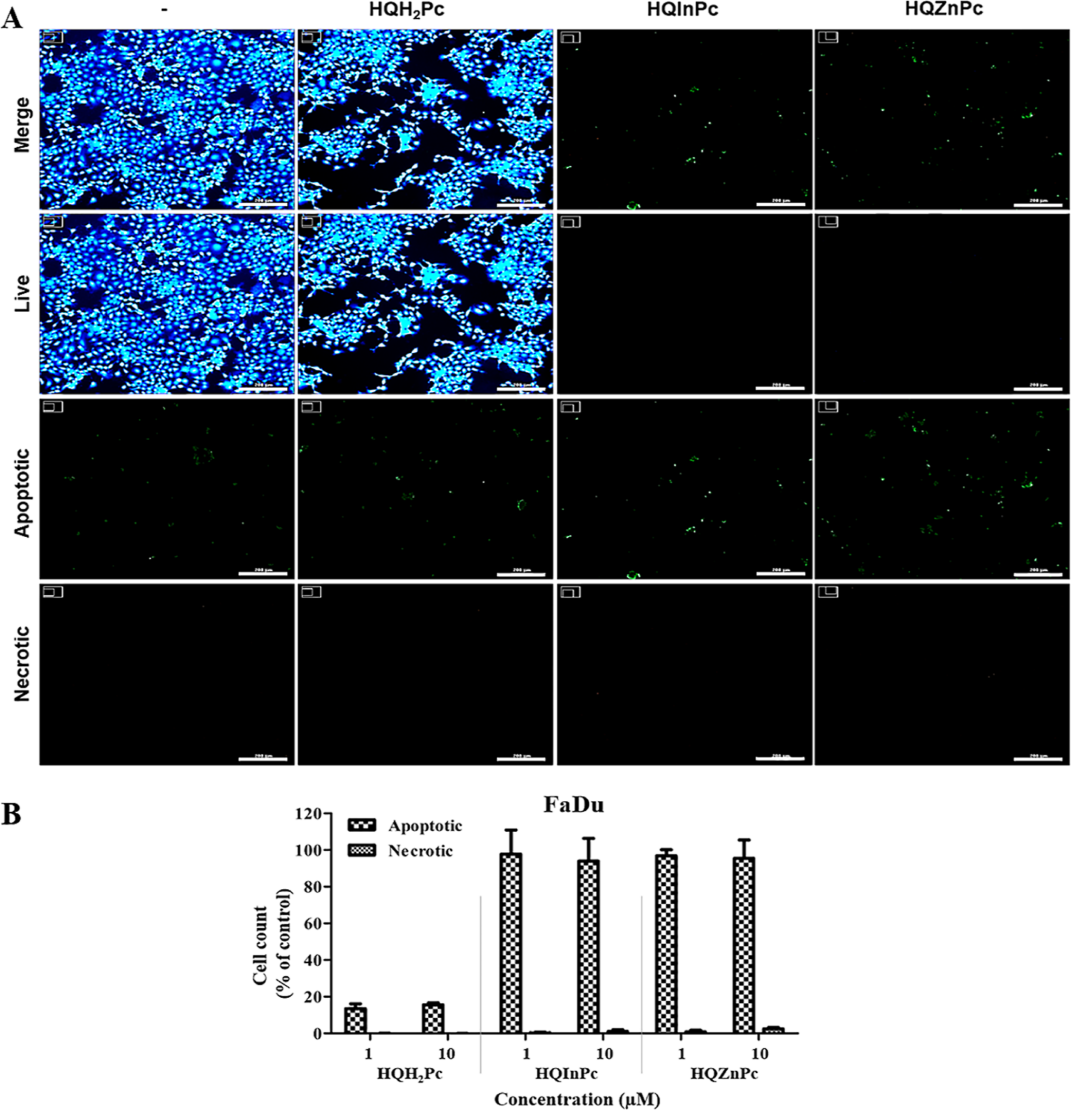
PDT-induced cellular death mechanism on FaDu cells mediated by **HQH**_**2**_ **Pc**, **HQInPc**, and **HQZnPc** molecules. (A) Fluorescence microscopy images of FaDu cells stained to identify live, apoptotic, and necrotic populations 24 h after PDT. Cells were treated with 10 μM HQH_2_Pc, HQInPc, or HQZnPc and irradiated with a light dose of 20 J/cm^2^. Live cells were stained with CytoCalcein Violet 450 (blue), apoptotic cells with Apoxin Green (green), and necrotic cells with 7-AAD (red). Scale bar: 200 μm. (B) Quantification of apoptotic and necrotic cell populations following treatment, presented as percentages relative to the total cell population.

**Figure 7:**
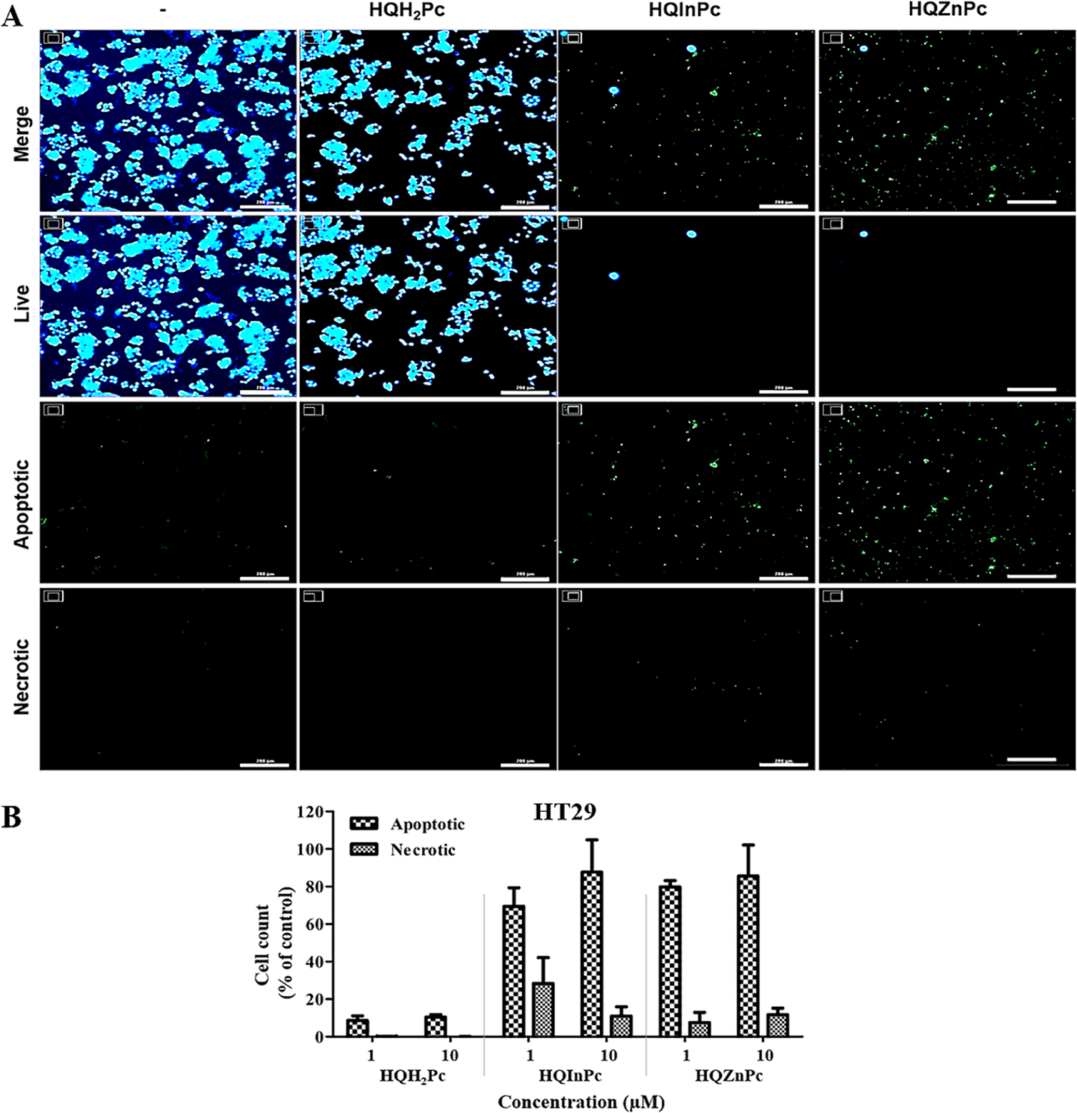
PDT-induced cellular death mechanism on HT29 cells mediated by **HQH**_**2**_ **Pc**, **HQInPc**, and **HQZnPc** molecules. (A) Fluorescence microscopy images of HT29 cells stained to identify live, apoptotic, and necrotic populations 24 h after PDT. Cells were treated with 10 μM HQH_2_Pc, HQInPc, or HQZnPc and irradiated with a light dose of 20 J/cm^2^. Live cells were stained with CytoCalcein Violet 450 (blue), apoptotic cells with Apoxin Green (green), and necrotic cells with 7-AAD (red). Scale bar: 200 μm. (B) Quantification of apoptotic and necrotic cell populations following treatment, presented as percentages relative to the total cell population.

Many factors, such as the degree of binding of Pc, their substituent groups, the nature of the central metal atom, and their aggregation state, significantly influence their absorption within the optical transmission window of biological tissues.^40,41^ Analysis of the cellular death mechanisms revealed that the nature of the central metal in Pcs with identical substituent groups plays a pivotal role in their effectiveness. Consistent with the literature, ZnPcs, despite varying substituents, consistently demonstrated superior results, attributable to the electronic configuration of the zinc­(II) ion.^38,42–44^ Compared to clinically established photosensitizers such as Photofrin, Verteporfin, and Temoporfin, which have been extensively studied for their PDT efficacy, the cationic Pc derivatives developed in this study exhibit promising *in vitro* profiles. 5-Aminolevulinic acid (ALA) is a highly tumor-specific photosensitizer precursor for PDT. IC_50_ values of 5-ALA have been reported in the range of 35–70 μM.^45,46^ Due to its hydrophilic nature, ALA exhibits limited cellular and tissue penetration, reducing its efficacy in treating deep or poorly vascularized tumors.^47^ Photofrin, a first-generation FDA-approved photosensitizer, typically demonstrates IC_50_ values ranging from 0.3 to 8 μM in various cancer cell lines.^48,49^ While effective and requiring relatively lower concentrations compared to 5-ALA to induce cytotoxicity, it is associated with limitations such as prolonged cutaneous photosensitivity due to its extended retention in tissues.^50^ Verteporfin and Temoporfin, second-generation photosensitizers, demonstrate higher PDT potency, often achieving IC_50_ values in the range of 0.5–2 μM in various cancer cell lines,^51,52^ but their clinical applications are hindered by poor aqueous solubility, rapid photobleaching, and limited selectivity.^53,54^ In contrast, HQZnPc displayed potent phototoxicity at a 1 μM concentration, resulting in over 90% cell death in FaDu cells, accompanied by elevated ROS levels and apoptosis. The morpholinoethoxy substituents and quaternized cationic nature of HQZnPc are likely to contribute to enhanced water solubility and cellular uptake, thus improving the pharmacodynamic properties relative to traditional agents. These findings underscore HQZnPc’s strong potential as a next-generation photosensitizer for PDT. To advance its clinical application, future studies should focus on improving the tumor selectivity and pharmacokinetics of **HQZnPc.** In addition to apoptosis and ROS-related mechanisms, alterations in cell cycle progression are also critical indicators of cellular responses to PDT. Although cell cycle analysis was not included in the current study, it represents a valuable approach for understanding whether photosensitizer-induced stress leads to specific cell cycle arrest or disruption. Given that many photosensitizers exert their cytotoxic effects by interfering with DNA replication or mitotic machinery,^55^ determining the precise phase at which these Pc derivatives exert their action may provide deeper mechanistic insight. Therefore, comprehensive cell cycle profiling will be an important component of future investigations, enabling a more complete characterization of the therapeutic effects and molecular mechanisms of these compounds. Investigating its subcellular localization will be pivotal for understanding its mechanism of action and achieving precise cancer cell targeting. The intracellular localization of photosensitizers is a critical determinant of PDT efficacy given the highly localized nature of ROS-mediated damage. Since ROS exhibit limited diffusion, their biological effects are strongly influenced by the subcellular compartments where photosensitizers accumulate. Several studies have reported that Zn­(II)-Pcs preferentially localize to mitochondria and lysosomes, organelles that play central roles in apoptotic signaling and cellular stress responses.^56–58^ This organelle-specific targeting has been associated with enhanced PDT-induced apoptosis and is thought to underlie the superior efficacy of Zn­(II)-Pcs relative to that of their metal-free counterparts. The amphiphilic and cationic structural modifications of HQZnPc suggest a high likelihood of mitochondrial and lysosomal targeting, as reported for similar compounds. Future work aiming to experimentally validate these hypotheses through colocalization studies using organelle-specific fluorescent markers may enable a more comprehensive understanding of the intracellular behavior of HQZnPc and its impact on PDT outcomes. Furthermore, the rational design of Pc derivatives with optimized substituent groups could enhance their tumor specificity and overall photodynamic efficiency. Although the present study demonstrates the promising potential of morpholinoethoxy-substituted cationic Pcs, particularly HQZnPc, in 2D *in vitro* cancer models, further investigations are warranted to enhance clinical translation. Future studies should focus on evaluating these compounds in three-dimensional (3D) tumor spheroid models, which better mimic the tumor microenvironment, including gradients of oxygen, nutrients, and cellular heterogeneity. Moreover, *in vivo* studies in suitable animal models are crucial to assess the pharmacokinetics, biodistribution, tumor selectivity, and systemic toxicity of HQZnPc. These advanced models will provide deeper insights into the therapeutic window, optimize light delivery strategies, and validate the long-term antitumor efficacy and safety profile required for potential clinical applications.

## 3 Conclusions

The PDT-induced cytotoxicity, ROS generation, and cell death mechanisms of cationic tetra-substituted metal-free (**HQH**_**2**_ **Pc**) and metallo-Pcs (**HQInPc** and **HQZnPc**), synthesized and characterized in accordance with the literature, were comprehensively studied. Neither the metal-free (**HQH**_**2**_ **Pc**) nor the metallo-Pcs (**HQInPc** and **HQZnPc**) exhibited cytotoxicity in the absence of light across the studied cell lines, confirming their nontoxic nature under dark conditions. Upon light activation, these Pcs demonstrated significant cytotoxicity, particularly in FaDu cells, underscoring their effectiveness as photosensitizers. Metallo-Pc-mediated PDT induced substantial ROS production in a dose- and time-dependent manner across all cell lines, strongly correlating with the cytotoxicity results, emphasizing the pivotal role of ROS in PDT. Furthermore, this study revealed that PDT with metallo-Pcs predominantly induced apoptosis as the primary cell death mechanism. The results from PDT-induced cytotoxicity and ROS generation demonstrate the promising potential of metallo-Pcs, particularly **HQZnPc**, for cancer therapy. To enhance the clinical applicability of Pcs, further studies should focus on optimizing ZnPc formulations, investigating subcellular localization, and exploring their mechanisms of action in more complex and clinically relevant tumor models.

## 4 Experimental Section/Methods

### 4.1 Materials

Human submaxillary salivary gland epidermoid carcinoma (A253), human colon colorectal adenocarcinoma (HT29), and human pharynx squamous carcinoma (FaDu) cells were purchased from the American Type Culture Collection (ATCC, United States). McCoy’s 5A modified medium, fetal bovine serum (FBS), Minimum Essential Medium Eagle with Joklik modification, Dulbecco’s phosphate buffered saline, and Hank’s balanced salt solution (HBSS) were purchased from Thermo Fisher Scientific (United States). 2′,7’ −Dichlorofluorescin diacetate (DCFDA), Hoechst 33342, and propidium iodide were purchased from Sigma-Aldrich (United States). The Apoptosis/Necrosis Detection Kit was purchased from Abcam (England).

### 4.2 Equipment

The reported ^1^H–NMR spectra were recorded on an Agilent VNMRS 500 MHz spectrometer. Mass spectra were measured on a Bruker Microflex LT MALDI-TOF MS spectrometer. IR spectra were recorded on a PerkinElmer One FT-IR spectrophotometer, and electronic spectra were recorded by using a Scinco LabProPlus UV/vis spectrophotometer. Spectral data are given in the Supporting Information file (Figures S1–S17).

## 5 Synthesis and Characterization of Phthalocyanines

Cationic metal-free (**HQH**_**2**_ **Pc**) and metallo-Pcs (**HQInPc** and **HQZnPc**) were synthesized according to the reported procedures.^35^ **HQH**_**2**_ **Pc**, **HQInPc**, and **HQZnPc** were obtained by the reaction of aliphatic nitrogen atoms in their neutral derivatives with methyl iodine. The synthesis steps are given below.

### 5.1 General Route for the Synthesis of Metal-Free and Metallo Phthalocyanine Derivatives

4-(2-Morpholinoethoxy)­phthalonitrile (0.3 g, 1.16 mmol) was dissolved in 1-pentanol (2.0 mL). Anhydrous metal salts (0.29 mmol) [no metal salt for metal-free Pc; 0.05 g Zn­(CH_3_COO)_2_ for zinc Pc and 0.06 g InCl_3_ for indium Pc] and a catalytic amount of DBU (1,8-diazabicyclo[5.4.0]­undec-7-ene) were added to the reaction medium. All reactions were carried out in a sealed glass tube (10 × 75 mm) under a nitrogen atmosphere at 145 °C for ca. 24 h. After cooling to room temperature, the suspension was precipitated with methanol/water (1:2 v/v), centrifuged, and washed with the same mixture and then dried in vacuo. The crude products (except indium Pc) were purified by column chromatography on alumina using THF: hexane (10:1) as eluent. Indium Pc was purified by washing with diethyl ether, hexane, cold methanol, and acetone.

#### 5.1.1 2­(3),9­(10),16­(17),23­(24)-Tetrakis­(2- morpholinoethoxy)­phthalocyanine

Yield: 0.11 g (37%). mp > 200 °C; Anal. Calcd for C_56_H_62_N_12_O_8_: C, 65.23; H, 6.06; N, 16.30%. Found: C, 65.30; H, 6.00; N, 16.60%. FT-IR (υ_max_, cm^–1^): 3289 (N–H), 3086 (Ar–H), 2920–2800 (Aliph-CH), 1604, 1454, 1428, 1322, 1276, 1230, 1113. ^1^H NMR (CDCl_3_): δ ppm: 7.74–7.65 (m, 12H, Ar–H), 4.33–4.31 (t, 8H, O–CH2), 3.97–3.95 (t, 16H, O–CH2), 3.09–3.06 (t, 8H, N–CH2), 2.85–2.82 (t, 16H, N–CH2), −4.55 (br s, 2H, NH). UV–vis (THF): λ_max_, nm (log ε): 330 (4.95), 667 (4.92), 703 (4.99). MS (MALDI-TOF): *m*/*z* 1032.78 [M + 1]^+^.

#### 5.1.2 2­(3),9­(10),16­(17),23­(24)-Tetrakis­(2- morpholinoethoxy)­phthalocyaninato zinc­(II)

Yield: 0.13 g (40%). mp > 200 °C; Anal. Calcd for C_56_H_60_N_12_O_8_Zn: C, 61.45; H, 5.53; N, 15.36%. Found: C, 61.50; H, 5.31; N, 14.99%. FT-IR (υ_max_, cm^–1^): 3063 (Ar–H), 2923–2800 (Aliph-CH), 1603, 1484, 1390, 1331, 1274, 1219, 1110. ^1^H NMR (CDCl_3_): δ ppm: 8.96 (br s, 4H–Ar–H), 8.53 (br s, 4H, Ar–H), 7.62–7.44 (m, 4H, Ar–H), 4.53 (br s, 8H, O–CH2), 3.78 (br s, 16H, O–CH2), 3.02 (br s, 8H, N–CH2), 2.71 (br s, 16H, N–CH2). UV–vis (THF): λ_max_, nm (log ε) 349 (5.27), 677 (5.62). MS (MALDI-TOF): *m*/*z* 1095.65 [M + 1]^+^.

#### 5.1.3 2­(3),9­(10),16­(17),23­(24)-Tetrakis­(2-morpholinoethoxy)­phthalacyaninato­(chloro)­indium­(III)

Yield: 0.13 g (39%). mp > 200 °C; Anal. Calcd. for C_56_H_60_ClInN_12_O_8_: C, 57.03; H, 5.13; N, 14.25%. Found: C, 56.80; H, 5.20; N, 14.52%. FT-IR (υ_max_, cm^–1^): 3059 (Ar–H), 2949–2848 (Aliph-CH), 1603, 1482, 1390, 1338, 1276, 1239, 1112. ^1^H NMR (CDCl_3_): δ ppm: 8.86–8.76 (d, 4H, Ar–H), 8.34–8.27 (d, 4H, Ar–H), 7.51–7.43 (d, 4H, Ar–H), 4.47 (br s, 8H, O–CH2), 3.89–3.86 (t, 16H, O–CH2), 3.06 (br s, 8H, N–CH2), 2.75 (br s, 16H, N–CH2). UV–vis (THF): λ_max_, nm (log ε) 358 (5.21), 700 (5.48). MS (MALDI-TOF): *m*/*z* 1180.16 [M + 1]^+^, 1141.76 [M-1-Cl]^+^.

### 5.2 General Route for the Synthesis of Quaternized Phthalocyanine Derivatives (HQH_2_Pc, HQInPc, and HQZnPc)

Metal-free Pc (0.1 g, 0.09 mmol) [or zinc Pc (0.1 g, 0.09 mmol) or indium Pc (0.1 g, 0.08 mmol)] was dissolved in 5 mL of chloroform, the excess methyl iodide (0.03 mL, 0.48 mmol) was added to this solution, and the reaction mixture was stirred under reflux for 4 h. After the room temperature was cooled, the resulting suspension was filtered off, washed successively with hot ethanol, ethyl acetate, THF, chloroform, hexane, and diethyl ether, and dried.

### 5.3 Quaternized Metal-Free Phthalocyanine (HQH_2_Pc)

Yield: 0.09 g (60%). mp > 200 °C; Anal. Calcd for C_60_H_74_I_4_N_12_O_8_: C, 45.07; H, 4.65; N, 10.51%. Found: C, 45.10; H, 4.31; N, 10.45%. FT-IR (υ_max_, cm^–1^): 3289 (N–H), 3010 (Ar–H), 2927–2870 (Aliph-CH), 1609, 1467, 1395, 1342, 1229, 1096. ^1^H NMR (DMSO-*d*_6_): δ ppm: 9.01–8.72 (br s, 8H, Ar–H), 7.88 (s, 4H, Ar–H), 5.13 (s, 8H, O–CH2), 4.42 (s, 8H, N–CH2), 4.20 (s, 16H, O–CH2), 4.00–3.84 (t, 16H, N–CH2), 3.60 (s, 12H, CH3). UV–vis (DMF): λ_max_, nm (log ε) 343 (4.64), 669 (4.81), 702 (4.84).

### 5.4 Quaternized Zinc Phthalocyanine (HQZnPc)

Yield: 0.12 g (75%). mp > 200 °C; Anal. Calcd for C_60_H_72_I_4_N_12_O_8_Zn: C, 43.35; H, 4.37; N, 10.11%. Found: C, 43.50; H, 4.11; N, 10.22%. FT-IR (υ_max_, cm^–1^): 3014 (Ar–H), 2944–2873 (Aliph-CH), 1603, 1465, 1391, 1334, 1221, 1091. ^1^H NMR (DMSO-*d*_6_): δ ppm: 9.41–9.36 (dd, 4H, Ar–H), 9.05–9.02 (d, 4H, Ar–H), 7.92 (s, 4H, Ar–H), 5.15 (s, 8H, O–CH2), 4.30 (s, 8H, N–CH2), 4.14 (s, 16H, O–CH2), 3.84–3.76 (d, 16H, N–CH2), 3.52 (s, 12H, CH3); UV–vis (DMF): λ_max_, nm (log ε): 357 (4.96), 678 (5.36).

### 5.5 Quaternized Indium Phthalocyanine (HQInPc)

Yield: 0.10 g (69%). mp > 200 °C; Anal. Calcd for C_60_H_72_ClI_4_InN_12_O_8_: C, 41.25; H, 4.15; N, 9.62%. Found: C, 41.00; H, 4.01; N, 9.75%. FT-IR (υ_max_, cm^–1^): 3000 (Ar–H), 2954–2874 (Aliph-CH), 1604, 1480, 1394, 1334, 1223, 1088. ^1^H NMR (DMSO-*d*_6_): δ ppm 9.43–9.42 (d, 4H, Ar–H), 9.10–9.04 (d, 4H, Ar–H), 7.97 (s, 4H, Ar–H), 5.16 (s, 8H, O–CH2), 4.30 (s, 8H, N–CH2), 4.14 (s, 16H, O–CH2), 3.84–3.76 (d, 16H, N–CH2), 3.54 (s, 12H, CH3); UV–vis (DMF): λ_max_, nm (log ε): 360 (4.92), 693 (5.23).

### 5.6 In Vitro PDT Activities of the ZnPcs

#### 5.6.1 Cell Culture

A253 and HT29 cells were cultured in McCoy’s 5A modified medium, supplemented with FBS (10%, v/v) and penicillin streptomycin (1%, v/v). FaDu cells were cultured in Minimum Essential Medium Eagle with Joklik modification supplemented with FBS (10%, v/v) and penicillin–streptomycin (1%, v/v).

#### 5.6.2 Photodynamic Therapy

Dark cytotoxicity assessments were carried out using a wide concentration range of Pc molecules (0.1–100 μM). Based on these results, the concentrations that showed no cytotoxic effects under dark conditions in the tested cell lines were selected for PDT assays. Therefore, the PDT efficacy was specifically evaluated at two representative concentrations within this nontoxic range to isolate light-induced effects.^59^ Following the administration of Pc molecules at two concentrations (1–10 μM), the cells were incubated in a humidified incubator (37 °C and 5% CO_2_) for 1 h to facilitate the cellular uptake of the Pc molecules. PDT application was conducted using ceLED (CETONI, Germany) equipment with a light dose of 20 J cm^–2^ at the appropriate wavelength (650–670 nm).

#### 5.6.3 Dark- and PDT-Induced Cytotoxicity

The cells were seeded onto 96-well plates (5 × 10^3^ cells/well), and cultured for 24 h in a humidified incubator (37 °C, 5% CO_2_). After incubation, varying concentrations of Pc molecules were administered to the cells and shielded from light. PDT group was subsequently exposed to 20 J cm^–2^ of radiation at 650–670 nm, then incubated in a humidified incubator (37 °C, 5% CO_2_). Following 24 h of treatment, cell viability was determined using the WST-1 assay.

### 5.7 Reactive Oxygen Species Production

PDT-induced ROS production in the cells was determined using 2′, 7′-DCFDA.^38^ The cells were seeded onto the wells of a 96-well plate (10^4^ cells/well) and incubated for 24 h (37 °C, 5% CO_2_). After, Pc molecules (1–10 μM) were administrated to the cells, PDT (20 J cm^–2^, 650–670 nm) was applied, and the cells were incubated for 30 min (immediate ROS production) and 24 h (delayed ROS production) in a humidified incubator (37 °C, 5% CO_2_). Following the incubation, the cells were treated with DCFDA (20 μM in HBSS), incubated for 30 min at 37 °C. ROS levels were measured quantitatively by recording the fluorescence emission at 529 nm (λ_ex = 504 nm) using a Cytation 5 microplate reader (Agilent, United States).

#### 5.7.1 Cellular Death Mechanism

PDT-induced cellular death mechanisms were determined using the Apoptosis/Necrosis Detection Kit (Abcam, Ab176749) according to the manufacturer’s instructions. The cells were seeded onto the wells of a 96-well plate (10^4^ cells/well) and incubated for 24 h (37 °C, 5% CO_2_). Afterward, Pc molecules (1–10 μM) were administered to the cells, PDT (20 J cm^–2^, 650–670 nm) was applied, and incubated for 24 h in a humidified incubator (37 °C, 5% CO_2_). Then, the cells were stained with CytoCalcein Violet 450, Apopxin Green, and 7-ADD, and incubated for 60 min, and images were taken using Cytation 5 (Agilent, United States). The quantitative analysis was carried out by counting the number of apoptotic and necrotic cells from the captured images using Gen5 software (Agilent, United States).

### 5.8 Statistics

Data are expressed as the mean ± SD. Comparison analysis between groups was performed using a two-way ANOVA test. GraphPad Software was used for statistical analysis. P values of <0.05 were considered statistically significant.

## Supplementary Material



## Data Availability

The data that support the findings of this study are available from the corresponding author upon request.

## Notes

FT-IR, ^1^H NMR, MALDI-TOF MS, and UV–vis spectra of metal-free, zinc, and indium phthalocyanines and their cationic derivatives (HQH_2_Pc, HQZnPc, and HQInPc) (PDF)
