# Supplementary material for: Morpholinoethoxy-Substituted Cationic Metal-Free and Metallo Phthalocyanines: In Vitro Photodynamic Therapy Activities, PDT-Induced ROS Level Measurements, and Cellular Death Mechanism
Source: ACS Bio Med Chem Au. 2025 Jun 26;5(4):766–77. doi: 10.1021/acsbiomedchemau.5c00137 (PMC12371505; doi:10.1021/acsbiomedchemau.5c00137)
Supplement: Supplementary file 1 [file bg5c00137_si_001.pdf]

SUPPLEMENTARY MATERIAL

Morpholinoethoxy substituted cationic metal-free and metallo phthalocyanines: in vitro photodynamic therapy activities, PDT-induced ROS level measurements, and cellular death mechanism

Muge Serhatli[1†], Seyma Isik[1,2†], Ayfer Kalkan[3], Mukaddes Özçeşmeci[3], Esin Hamuryudan[3], Özge Can[4*]*

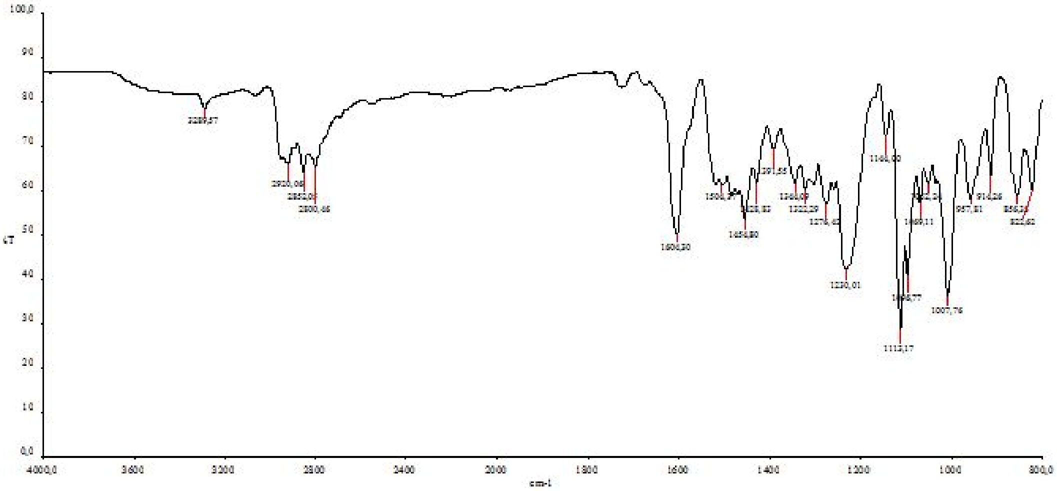

Figure S1. FT-IR spectrum of metal-free phthalocyanine.

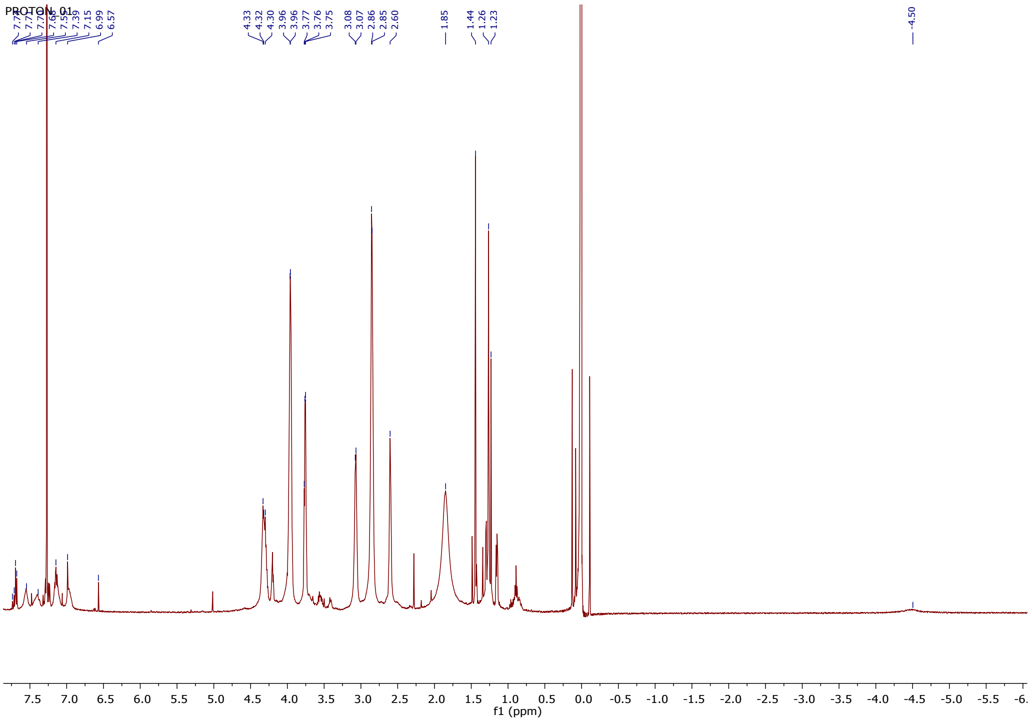

Figure S2. ^{1}H NMR spectrum of metal-free phthalocyanine.

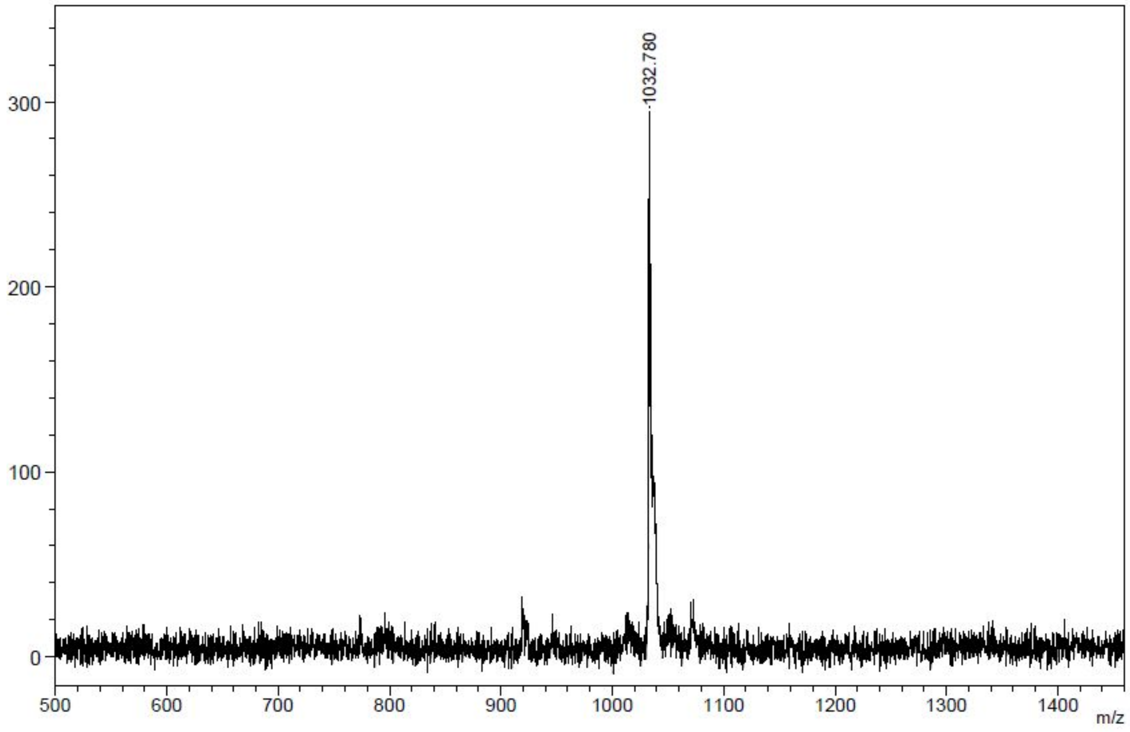

Figure S3. MALDI-TOF MS spectrum of metal-free phthalocyanine.

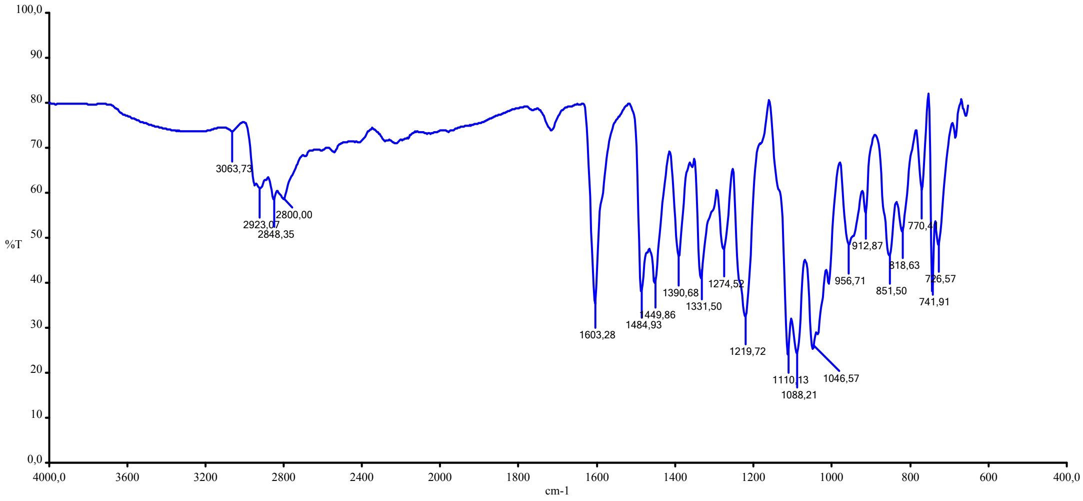

Figure S4. FT-IR spectrum of zinc phthalocyanine.

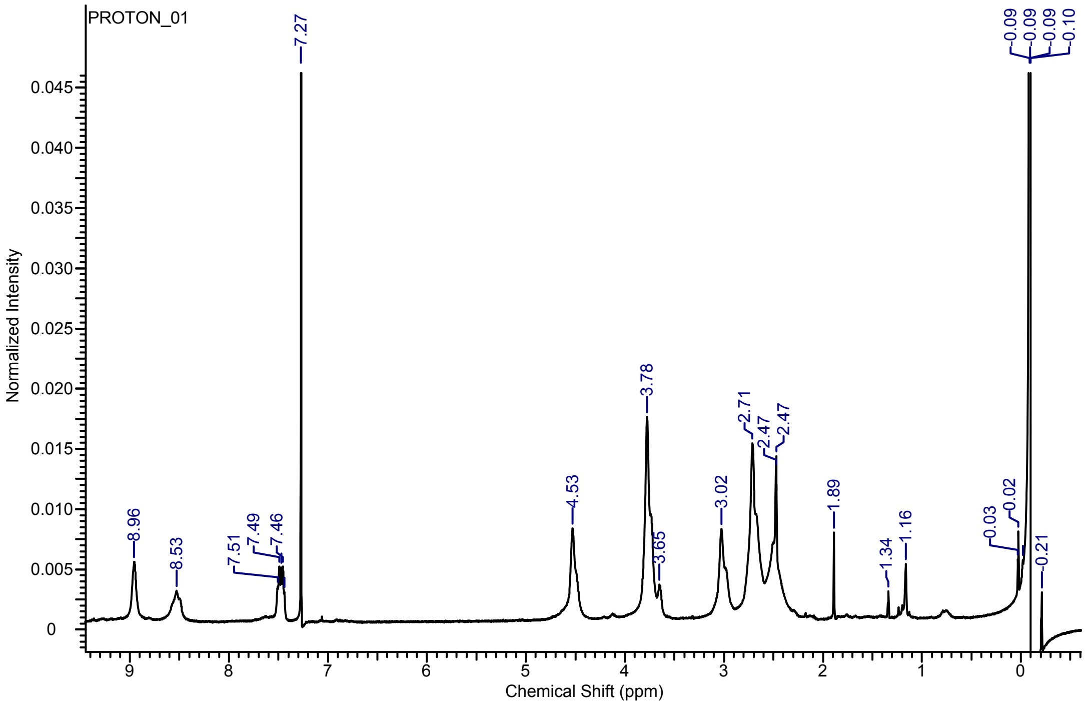

Figure S5. ^{1}H NMR spectrum of zinc phthalocyanine.

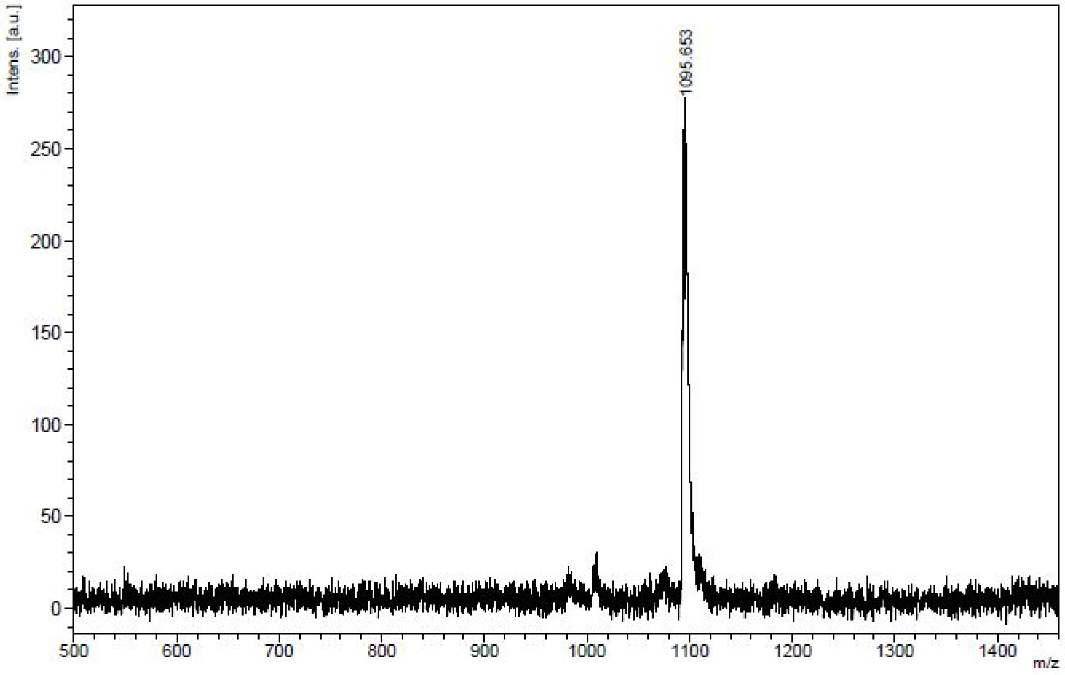

Figure S6. MALDI-TOF MS spectrum of zinc phthalocyanine.

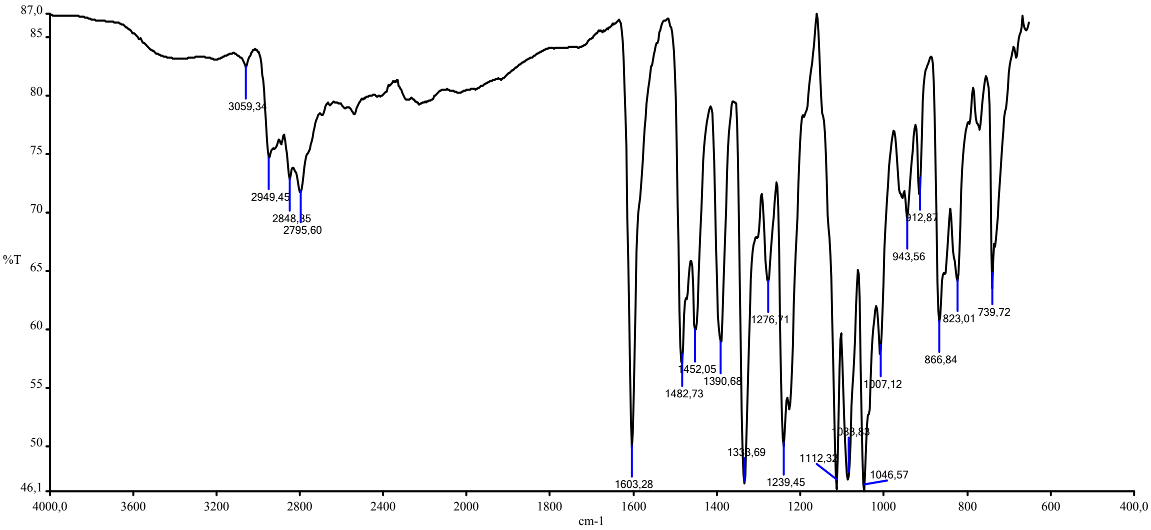

Figure S7. FT-IR spectrum of indium phthalocyanine.

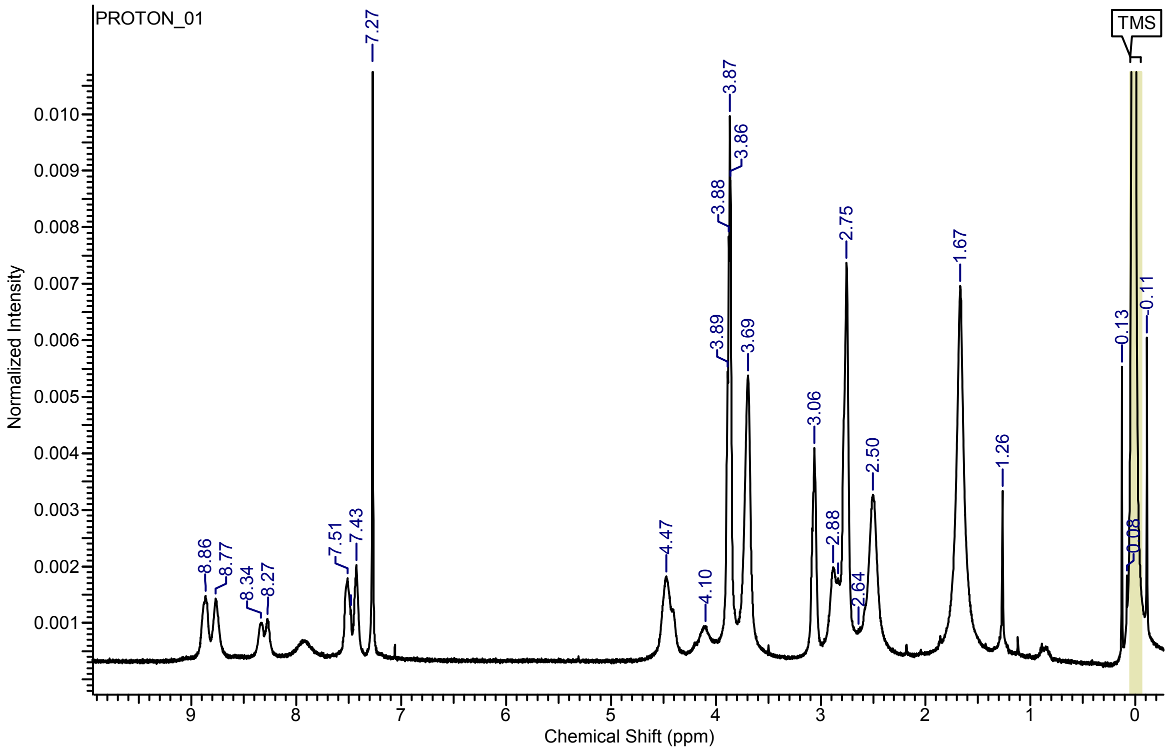

Figure S8. ^{1}H NMR spectrum of indium phthalocyanine.

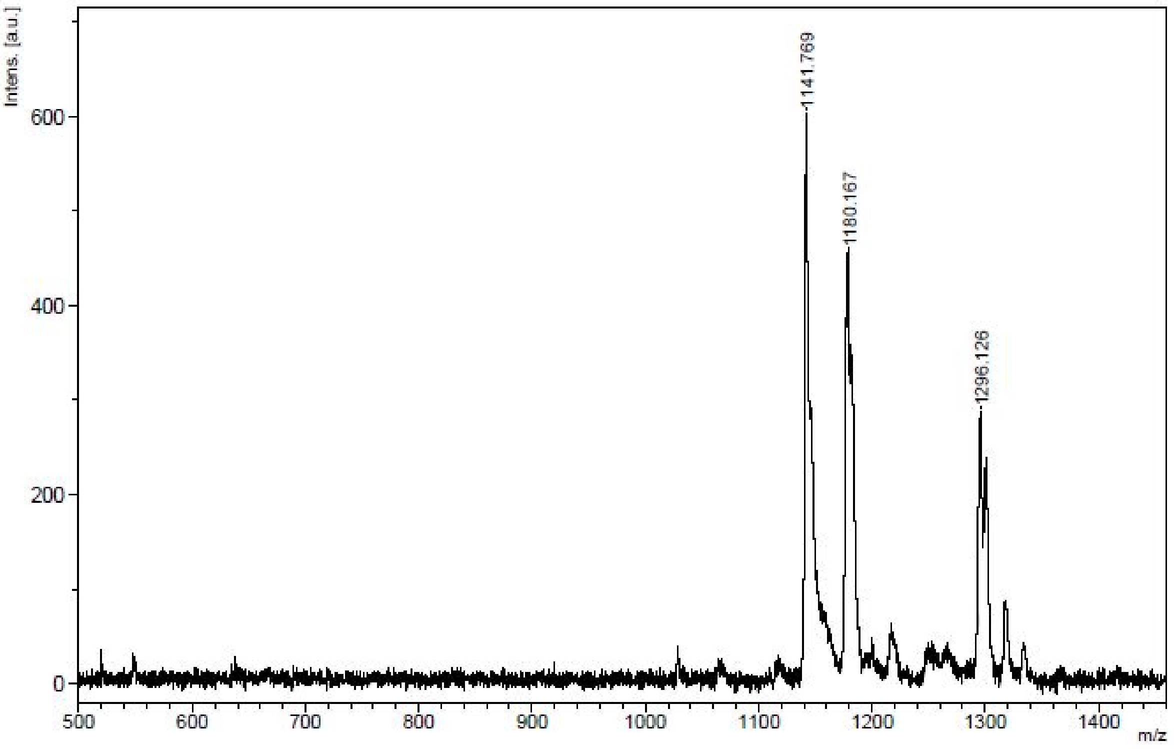

Figure S9. MALDI-TOF MS spectrum of indium phthalocyanine.

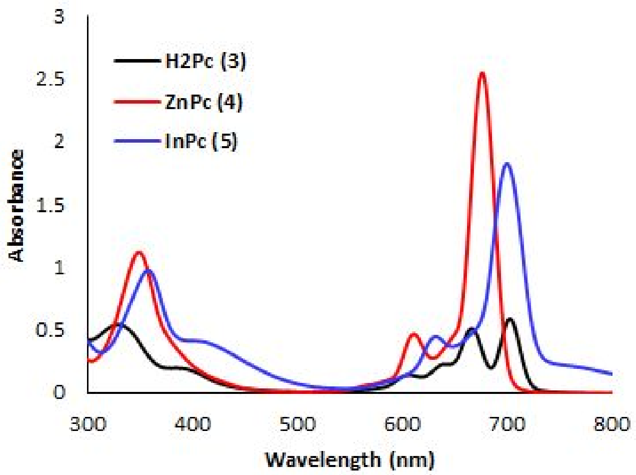

Figure S10. UV-Vis absorption spectra of metal-free (**3**), zinc (**4**) and indium (**5**) phthalocyanines in THF (6 x 10^{-6} M)

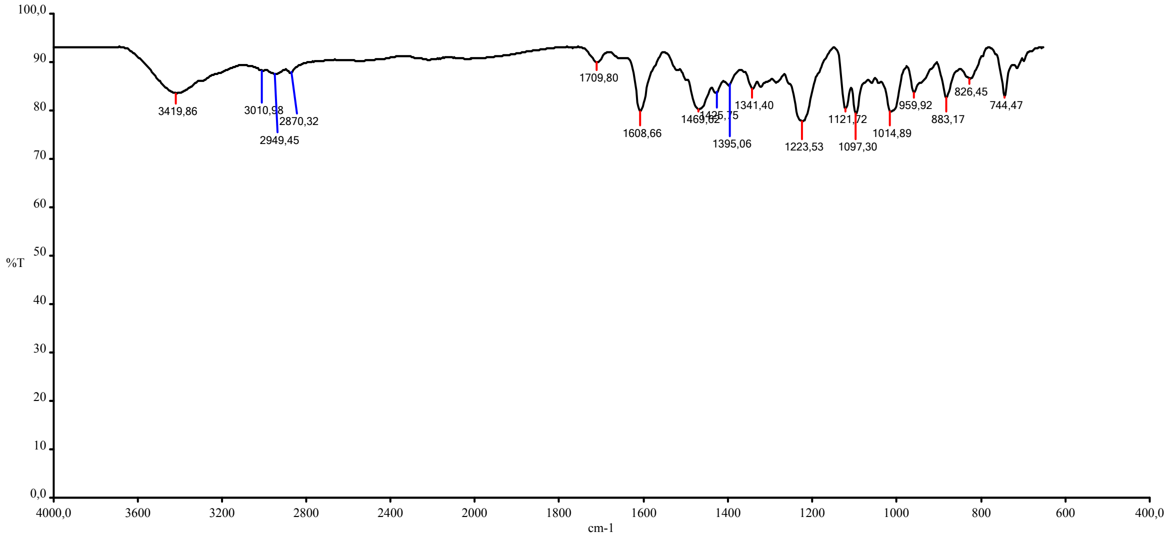

Figure S11. FT-IR spectrum of cationic metal-free phthalocyanine (**HQH_2Pc**).

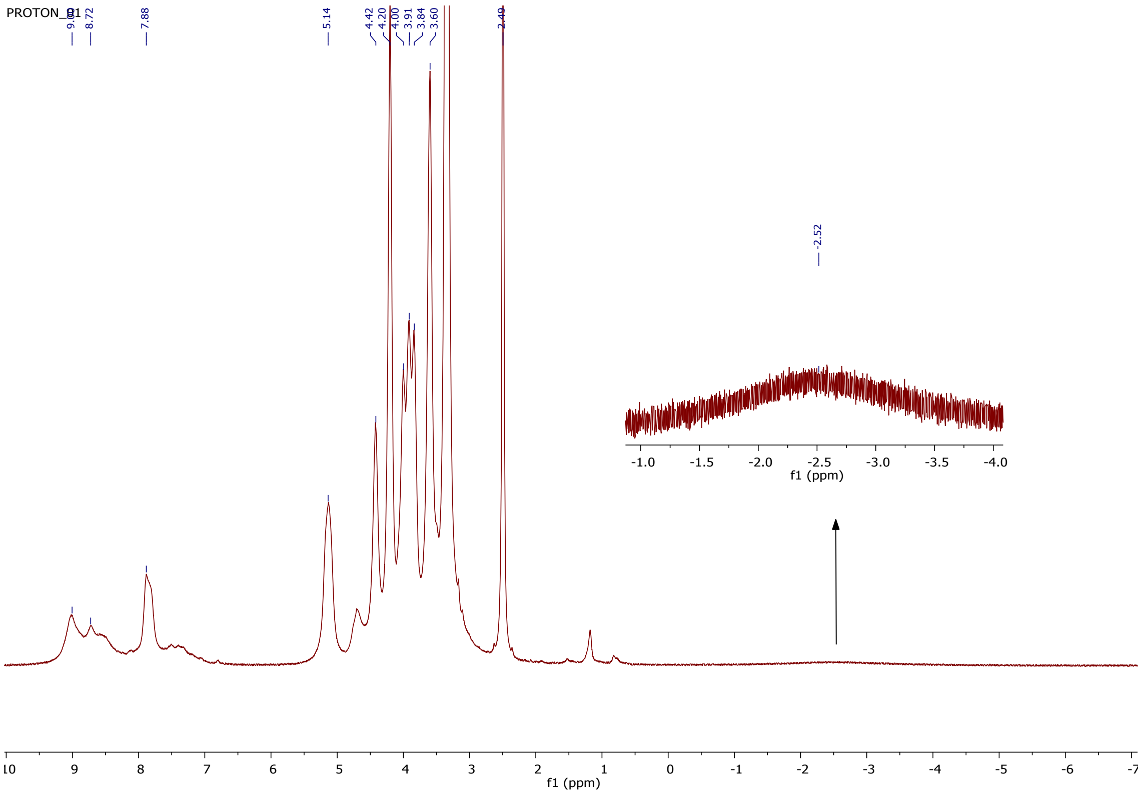

Figure S12. ^{1}H NMR spectrum of cationic metal-free phthalocyanine (**HQH_2Pc**).

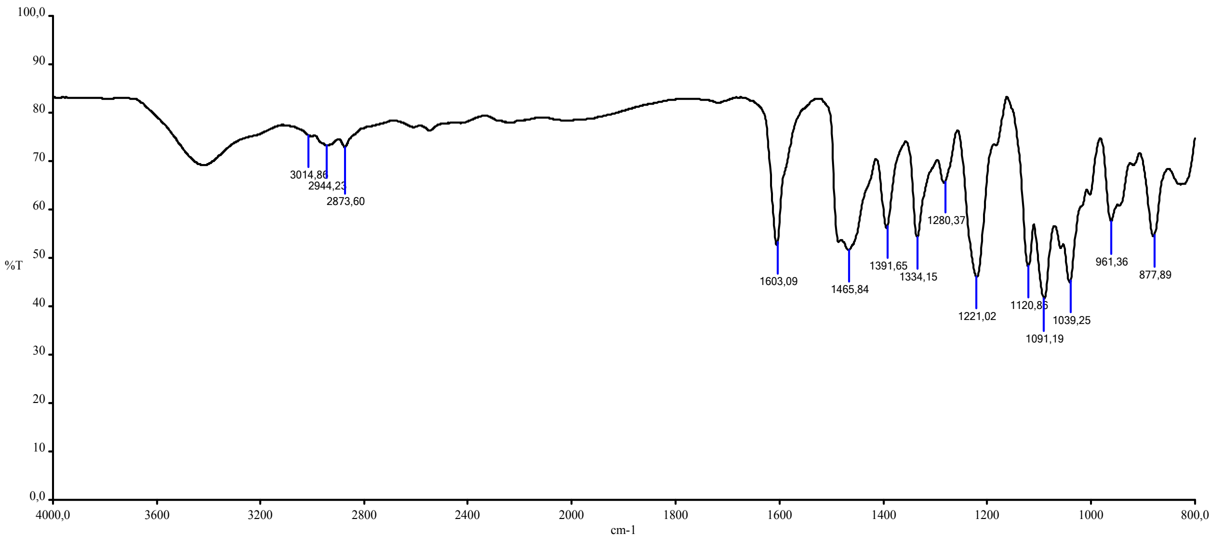

Figure S13. FT-IR spectrum of cationic zinc phthalocyanine (**HQZnPc**).

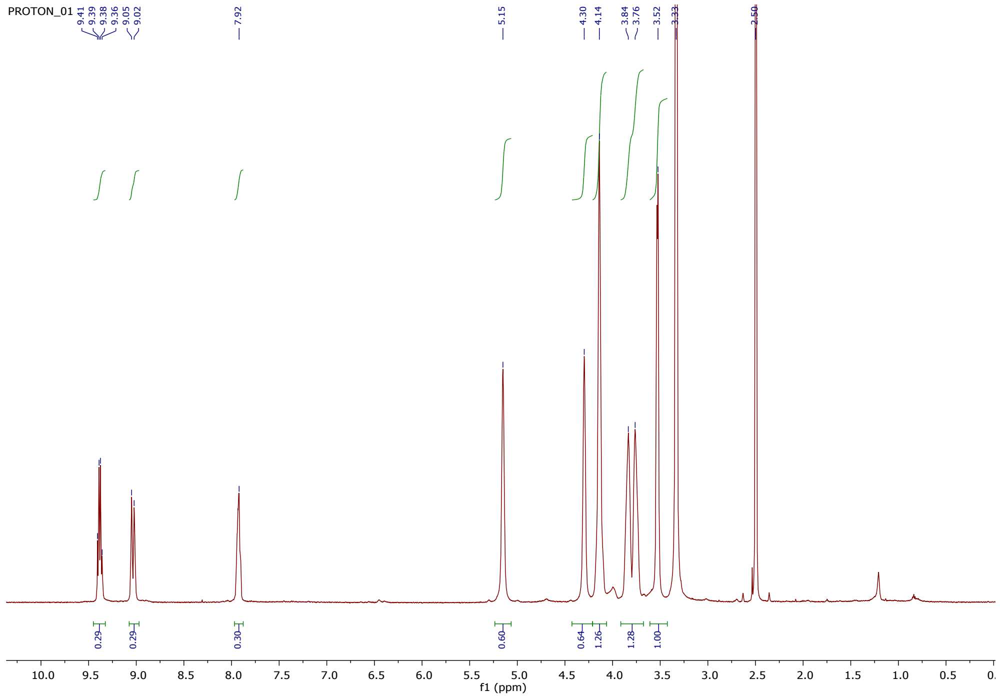

Figure S14. ^{1}H NMR spectrum of cationic zinc phthalocyanine (**HQZnPc**).

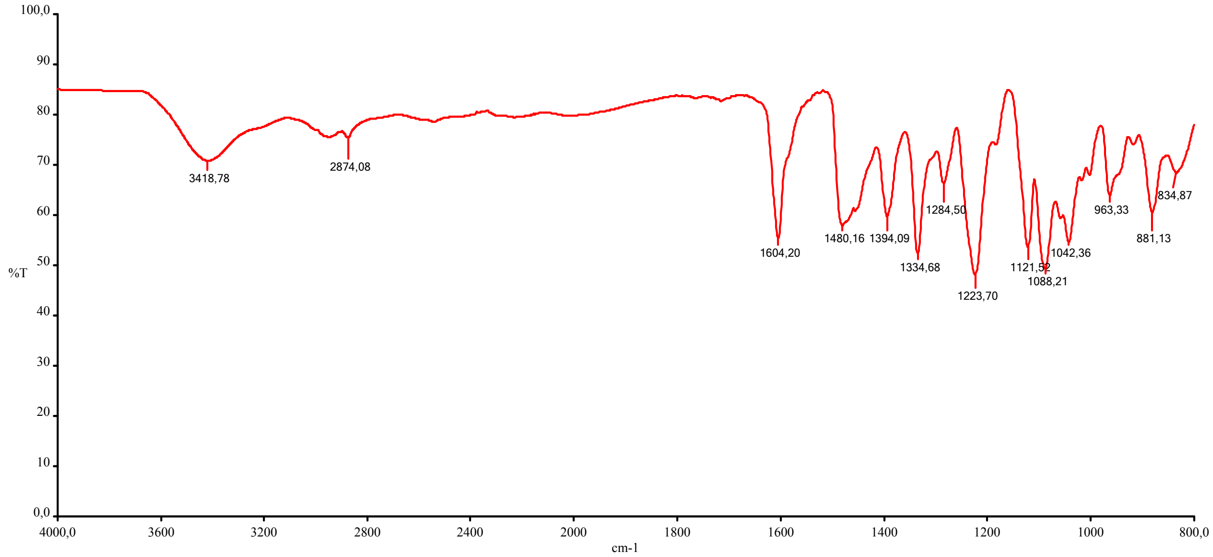

Figure S15. FT-IR spectrum of cationic indium phthalocyanine (**HQInPc**).

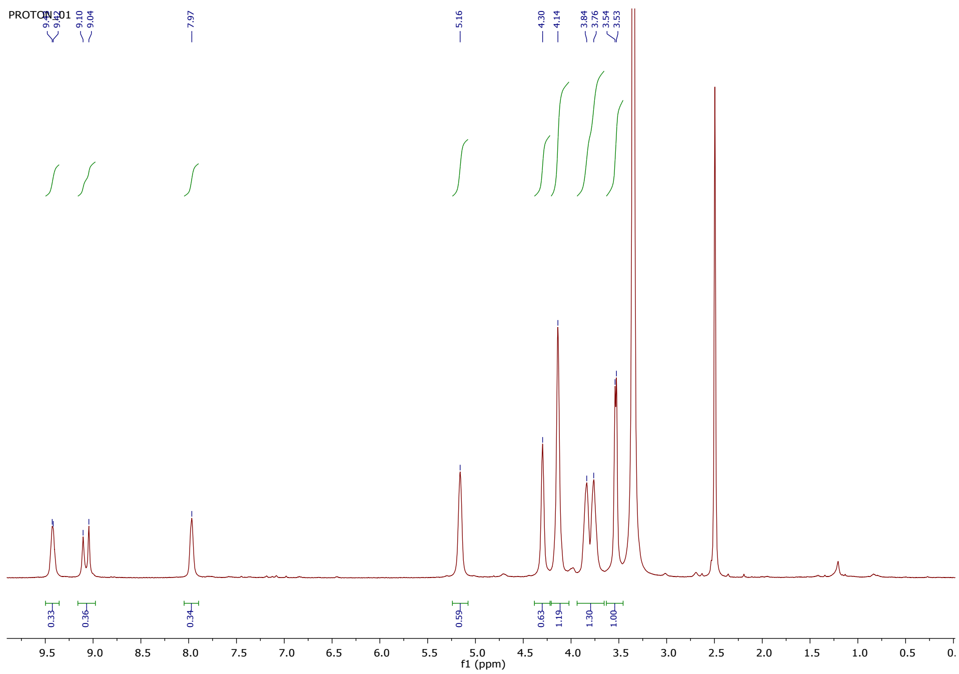

Figure S16. ^{1}H NMR spectrum of cationic indium phthalocyanine (**HQInPc**).

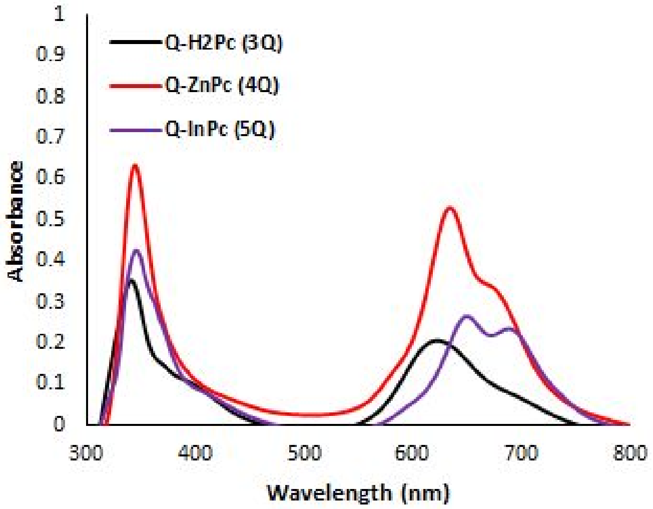

Figure S17. UV-Vis absorption spectra of **HQH$_2$Pc (3Q), HQZnPc (4Q),** and **HQInPc (5Q)** in water (1 x 10^{-5} M)
